# Fuzuloparib with or without apatinib in patients with HER2-negative metastatic breast cancer with germline BRCA1/2 mutations: a cost-utility analysis

**DOI:** 10.3389/fphar.2026.1897783

**Published:** 2026-07-29

**Authors:** Yitian Lang, Meng Deng, Jie Yang

**Affiliations:** 1 Department of Pharmacy, Shanghai Second People’s Hospital, Shanghai, China; 2 Department of Pharmacy, Tongren Hospital, Shanghai Jiao Tong University School of Medicine, Shanghai, China

**Keywords:** apatinib, cost-utility, fuzuloparib, germline BRCA1/2 mutations, HER2-negative breast cancer, partitioned survival approach

## Abstract

**Background:**

Fuzuloparib, a novel PARP inhibitor, has demonstrated efficacy in patients with HER2-negative metastatic breast cancer (MBC) harboring germline BRCA1/2 mutations. The addition of apatinib, an anti-angiogenic agent, enhances treatment outcomes. However, the economic value of these strategies remains unclear.

**Objective:**

To evaluate the cost-utility of fuzuloparib with or without apatinib compared with standard chemotherapy in patients with HER2-negative metastatic breast cancer with germline BRCA mutations from the Chinese healthcare system perspective.

**Methods:**

A partitioned survival model was developed over a 10-year time horizon. Clinical inputs were derived from the phase III FABULOUS trial. Survival data were reconstructed and extrapolated using parametric models. Costs and utilities were obtained from published literature and Yaozh databases. Outcomes were measured in quality-adjusted life years (QALYs) and incremental cost-utility ratios (ICURs). A willingness-to-pay (WTP) threshold of ¥199,330/QALY was applied. Deterministic and probabilistic sensitivity analyses were conducted to assess model uncertainty.

**Results:**

In the base-case analysis, total costs were ¥266,989.27 for fuzuloparib plus apatinib (FA), ¥230,151.42 for fuzuloparib monotherapy (FM), and ¥188,774.18 for standard chemotherapy (SC). Corresponding QALYs were 1.5669, 1.4128, and 1.1477, respectively. Compared with SC, the ICURs were ¥186,581.80 per QALY for FA and ¥156,081.63 per QALY for FM, both lower than the WTP threshold. Compared with FM, the ICUR was ¥239,051.59 per QALY for FA. Sensitivity analyses confirmed that the probability of the FA, FM and SC being cost-effective were 29.15%, 42.72%, and 28.13% at the WTP threshold.

**Conclusion:**

From the perspective of the Chinese healthcare system, both fuzuloparib plus apatinib and fuzuloparib monotherapy is cost-effective compared with standard chemotherapy at current WTP threshold. However, FA was not cost-effective relative to FM at the current Chinese WTP threshold.

## Introduction

Breast cancer remains the most commonly diagnosed cancer worldwide and a leading cause of cancer-related mortality among women. According to the latest global cancer statistics, more than 2.3 million new cases of breast cancer were diagnosed in 2022, accounting for approximately 11.6% of all newly diagnosed cancers globally, and nearly 0.66 million deaths were attributed to this disease (Bray et al., 2024). Despite substantial advances in early detection and treatment, metastatic breast cancer (MBC) continues to be largely incurable, with treatment strategies primarily aiming to prolong survival, control symptoms, and maintain quality of life (Cardoso et al., 2020).

Human epidermal growth factor receptor 2 (HER2)-negative breast cancer accounts for around 80% of breast cancer cases, including both hormone receptor–positive and triple-negative subtypes (Giaquinto et al., 2024). A clinically important subgroup of patients with HER2-negative breast cancer carries germline mutations in the BRCA1 or BRCA2 genes. These mutations impair homologous recombination repair of DNA double-strand breaks, leading to genomic instability and increased susceptibility to tumor development (Lord and Ashworth, 2016). Approximately 5%–10% of patients with HER2-negative breast cancer carry germline BRCA mutations, and these patients often present with more aggressive disease and distinct therapeutic vulnerabilities (O’Shaughnessy et al., 2020).

The discovery of synthetic lethality between BRCA deficiency and inhibition of poly (ADP-ribose) polymerase (PARP) has led to the development of PARP inhibitors as a targeted treatment strategy for BRCA-mutated cancers. PARP enzymes play a critical role in repairing single-strand DNA breaks; inhibition of PARP results in accumulation of DNA damage and eventual tumor cell death in BRCA-deficient cells (Lord and Ashworth, 2017). Clinical evidence supporting the efficacy of PARP inhibitors in breast cancer has been demonstrated in several randomized controlled trials. The phase III OlympiAD trial showed that olaparib significantly improved progression-free survival compared with standard chemotherapy in patients with HER2-negative metastatic breast cancer harboring germline BRCA mutations (Robson et al., 2017). Similarly, the EMBRACA trial demonstrated that talazoparib was associated with longer progression-free survival and better patient-reported outcomes compared with physician’s choice chemotherapy (Litton et al., 2018). These findings established PARP inhibitors as an important treatment option for this patient population and led to regulatory approvals in multiple regions.

Despite these advances, treatment outcomes for metastatic breast cancer remain suboptimal, and resistance to PARP inhibitors may develop over time. Therefore, strategies to enhance the efficacy of PARP inhibition have become an area of active research. One promising approach involves combining PARP inhibitors with anti-angiogenic agents. Tumor angiogenesis, primarily mediated through vascular endothelial growth factor (VEGF) signaling pathways, plays a crucial role in tumor growth and metastasis. Anti-angiogenic therapies can modify the tumor microenvironment, reduce hypoxia, and potentially influence DNA repair pathways. Preclinical studies have suggested that anti-angiogenic therapy downregulate homologous recombination repair mechanisms, thereby increasing sensitivity to PARP inhibition and enhancing antitumor activity (Bindra et al., 2005; Chan et al., 2010).

Fuzuloparib is a novel, orally administered PARP1/2 inhibitor that has demonstrated potent inhibition of PARP activity and promising antitumor effects in preclinical and early clinical studies (Wang et al., 2019). In parallel, apatinib is a highly selective tyrosine kinase inhibitor targeting VEGFR2, which has shown anti-angiogenic activity and clinical benefits in several solid tumors (Zhao et al., 2018). Recently, a randomized phase III clinical trial evaluated fuzuloparib with or without apatinib compared with chemotherapy in patients with HER2-negative metastatic breast cancer harboring germline BRCA1/2 mutations, demonstrating significant improvements in progression-free survival with PARP inhibitor–based regimens (Li et al., 2025).

While clinical efficacy is a critical consideration, the rapid development and adoption of targeted therapies have raised substantial concerns regarding healthcare costs and economic sustainability. Novel anticancer drugs, particularly targeted agents and combination therapies, are often associated with high prices, which may impose a significant financial burden on healthcare systems and patients. Economic evaluation has therefore become an essential component of healthcare decision-making, helping policymakers determine whether new interventions provide sufficient value relative to their costs. Cost-utility analysis is widely used to assess the value of new cancer treatments and inform reimbursement and pricing policies (Bertram et al., 2021).

Given the encouraging clinical outcomes of PARP inhibitor–based therapy observed in recent trials and the increasing availability of these agents in clinical practice, it is crucial to evaluate their economic implications. In particular, the combination of a PARP inhibitor with an anti-angiogenic agent further increases treatment costs, making cost-utility analysis even more relevant. However, economic evidence for fuzuloparib alone or in combination with apatinib remains limited. Therefore, the present study aimed to conduct a cost-utility analysis of fuzuloparib with or without apatinib compared with standard chemotherapy for patients with HER2-negative metastatic breast cancer with germline BRCA1/2 mutations from the Chinese healthcare system perspective, using clinical outcomes derived from FABULOUS trial. This study provides evidence to support clinical decision-making, resource allocation, and policy development regarding the optimal use of PARP inhibitor–based therapies in this patient population.

## Methods and materials

The Consolidated Health Economic Evaluation Reporting Standards (CHEERS) offers a complete set of guidelines designed to boost the transparency and replicability of health economic assessment studies (Husereau et al., 2022). The present economic evaluation adhered to the CHEERS standards, as detailed in Supplementary Table S1.

### Model structure

**FIGURE 1:**
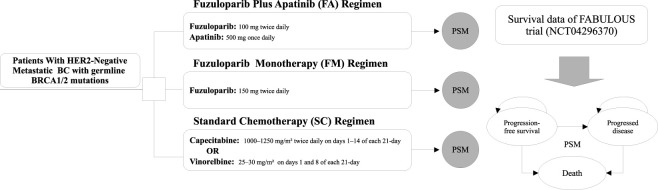
The decision tree and partitioned survival model structure overview. Abbreviations: HER2 human epidermal growth factor receptor-2, BC breast cancer, PSM partitioned survival model**.**

An economic evaluation model was developed to assess the cost-utility of fuzuloparib, administered either as monotherapy or in combination with apatinib, in comparison with standard chemotherapy. The analysis was conducted using a partitioned survival model (PSM), a modeling framework frequently applied in oncology economic evaluations to capture disease progression over time. The model simulated the clinical course of patients with HER2-negative metastatic breast cancer harboring germline BRCA1/2 mutations and comprised three mutually exclusive health states: progression-free (PF), progressed disease (PD), and death. At model initiation, all patients were assumed to enter the PF state. Over time, patients could remain PF or transition to the PD or death states, with transitions determined implicitly from survival outcomes reported in the clinical trial. Within the PSM approach, the distribution of patients across health states at each time point is estimated directly using the progression-free survival (PFS) and overall survival (OS) curves. A 3-week model cycle length was used to align with the administration schedule of standard chemotherapy regimen. A 10-year time horizon was adopted to ensure that more than 90% of the simulated cohort entered the absorbing death state, thereby capturing nearly all lifetime costs and health outcomes relevant to the study population. The decision tree and PSM structure are shown in Figure 1.

### Treatment regimens and resource use

This analysis incorporated treatment regimens based on the FABULOUS trial, including three interventions: (1) fuzuloparib plus apatinib (FA) regimen and (2) fuzuloparib monotherapy (FM) regimen and (3) standard chemotherapy (SC) regimen. The dosing strategies for all arms were also in line with those used in the FABULOUS trial. Therein, fuzuloparib was administered orally at 100 mg twice daily, and apatinib at 500 mg once daily in the FA arm. Fuzuloparib was administered orally at 150 mg twice daily in the FM arm. In the SC arm, treatment consisted of either capecitabine 1000–1250 mg/m^2^ administered orally twice daily on days 1–14 of each 21-day cycle, or vinorelbine 25–30 mg/m^2^ intravenously on days 1 and 8 of each 21-day cycle.

In all treatment arms, therapy was continued until disease progression or unacceptable toxicity. Subsequent systemic anti-tumor therapy was defined according to the treatment patterns reported in the FABULOUS trial, and all regimens incorporated into the model were consistent with the recommendations of the Chinese Society of Clinical Oncology (CSCO) guidelines for HER2-negative metastatic breast cancer. According to the FABULOUS trial, subsequent treatment was received by 42.9%, 55.2%, and 60.6% of patients in the FA, FM, and SC groups, respectively (Li et al., 2025). Given the diversity of subsequent treatment options reported in the trial and the lack of patient-level information regarding the exact sequence and duration of multiple subsequent treatment lines, a weighted-average costing approach was adopted. Specifically, chemotherapy, endocrine therapy, immunotherapy, and targeted therapy were included in the model, whereas other therapies were excluded because of their low frequency and heterogeneous composition. The proportion of each treatment category among patients receiving subsequent therapy in each study arm was calculated based on the FABULOUS trial, and the corresponding subsequent treatment costs were estimated as weighted average costs according to these proportions. Patients may receive more than one category of subsequent therapy. Therefore, proportions are not mutually exclusive. Representative regimens recommended by the CSCO guidelines were selected for each treatment category, including SC regimen for chemotherapy, palbociclib plus anastrozole for endocrine therapy, toripalimab plus nab-paclitaxel for immunotherapy, and fuzuloparib monotherapy for targeted therapy. Therefore, the expected subsequent treatment cost for each study arm was calculated using a two-stage weighted approach that accounted for both the probability of receiving subsequent treatment and the distribution of different treatment categories among treated patients. The detailed composition of subsequent treatment categories, representative regimens, and the weighting procedures used to estimate subsequent treatment costs for each study arm are provided in Supplementary Table S2.

### Clinical data

Key clinical inputs, including PFS, OS data and incidence of severe adverse events (AEs) were sourced from the FABULOUS trial (Li et al., 2025). Since the duration of trial follow-up was insufficient to cover the full analytic horizon of the PSM, long-term survival outcomes were extrapolated beyond the observed data. In principle, survival extrapolation requires individual patient data (IPD) to enable parametric modeling. As IPD were not publicly accessible, pseudo-IPD were reconstructed from published Kaplan–Meier curves using the algorithm proposed by Guyot et al. (2012). This method has been widely applied in survival analyses and economic evaluations when original patient-level data are inaccessible (Saluja et al., 2019). The reconstructed IPD were subsequently fitted to several commonly used parametric survival distributions, including Weibull, log-logistic, log-normal, exponential, Gompertz, and gamma distributions (Jackson, 2016). Model selection was based on statistical goodness-of-fit criteria, including the Akaike information criterion (AIC) and Bayesian information criterion (BIC), together with visual inspection of the fitted curves.

Adverse events associated with treatment were incorporated into the analysis. Considering that grade 1 to 2 AEs are typically manageable and have limited impact on costs or quality of life, only grade 3 and higher events were included. The parameters of the selected survival distributions and the incidence of severe AEs are summarized in Table 1.

**TABLE 1:** Key clinical data.

Parameters	Estimated values	Range	Distribution
PFS: FA group	meanlog = 2.6817 sdlog = 0.8871	meanlog (2.4473–2.9162) sdlog (0.7244–1.0862)	Log-normal
OS: FA group	meanlog = 3.73 sdlog = 0.885	meanlog (3.424–4.036) sdlog (0.661–1.184)	Log-normal
PFS: FM group	meanlog = 2.198 sdlog = 0.962	meanlog (1.951–2.445) sdlog (0.788–1.175)	Log-normal
OS: FM group	meanlog = 3.683 sdlog = 0.844	meanlog (3.377–3.989) sdlog (0.618–1.153)	Log-normal
PFS: SC group	meanlog = 1.5854 sdlog = 0.8627	meanlog (1.355–1.8157) sdlog (0.7056–1.0548)	Log-normal
OS: SC group	meanlog = 3.385 sdlog = 1.049	meanlog (3.031–3.739) sdlog (0.794–1.385)	Log-normal
Risk of main grade 3 and more AEs in the FA group			
Decreased white blood cells	8.57%	6.43%–10.71%	Beta
Neutrophil count decreased	12.86%	9.65%–16.08%	Beta
Anemia	11.43%	8.57%–14.29%	Beta
Platelet count decreased	12.86%	9.65%–16.08%	Beta
Risk of main grade 3 and more AEs in the FM group			
Decreased white blood cells	19.40%	14.55%–24.25%	Beta
Neutrophil count decreased	20.90%	15.68%–26.13%	Beta
Anemia	37.31%	27.98%–46.64%	Beta
Platelet count decreased	11.94%	8.96%–14.93%	Beta
Risk of main grade 3 and more AEs in the SC group			
Decreased white blood cells	18.64%	13.98%–23.30%	Beta
Neutrophil count decreased	23.73%	17.80%–29.66%	Beta
Anemia	8.47%	6.35%–10.59%	Beta
Platelet count decreased	5.08%	3.81%–6.35%	Beta
Proportions of patients receiving subsequent treatment			
FA group	42.9%	32.175%–53.625%	Beta
FM group	55.2%	41.4%–69%	Beta
SC group	60.6%	45.45%–75.75%	Beta

Abbreviations: FA, fuzuloparib plus apatinib; FM, fuzuloparib monotherapy; SC, standard chemotherapy; AEs, adverse events; PFS, progression-free survival; OS, overall survival.

### Costs and utilities

The economic evaluation was conducted from the perspective of the Chinese healthcare system and included only direct medical expenditures. These costs encompassed several components, including the acquisition of anticancer medications, drug administration, follow-up, palliative care, and the management of treatment-related adverse events (AEs) of grade 3 or higher. Follow-up expenses primarily consisted of routine imaging examinations, diagnostic tests, and disease monitoring during the treatment period. Drug acquisition costs were obtained from the Yaozh database (Chinese Drug Price of Drug Centralized Bid Procurement., 2026). For fuzuloparib and apatinib, the official listed prices in 2026 were used because both drugs are proprietary products. For capecitabine and vinorelbine, multiple bid-winning prices from different manufacturers were available in the database; therefore, the median bid-winning price in 2026 was adopted to better reflect the average procurement price in routine clinical practice in China. Other medical costs, such as those related to intravenous drug administration, palliative care services, follow-up visits, and subsequent lines of therapy, were derived from published studies or estimated using available cost data (Lang et al., 2024; Chinese Drug Price of Drug Centralized Bid Procurement., 2026). In addition, the costs associated with severe treatment-related AEs were incorporated into the model based on the incidences reported in the FABULOUS trial. The costs of managing severe AEs were extracted from previously published literature (Kang et al., 2026; Zhu et al., 2026). Healthcare resource costs extracted from published literature were adjusted using the healthcare component of the Consumer Price Index (CPI) reported in the China Statistical Yearbook.

Health utility parameters were incorporated into the model to estimate total quality-adjusted life years (QALYs), which capture health-related quality of life (HRQoL) over the disease course. The utility values were assigned to the PF and PD health states. In the absence of trial-reported utility data, these values were obtained from previously published studies, which reported the health utility outcomes for patients receiving PARP inhibitor for HER2-negative metastatic breast cancer with germline BRCA1/2 mutations (Schwarz et al., 2022). The utility decrements associated with severe AEs were incorporated into the model. And the data were extracted from published literature (Kang et al., 2026; Zhu et al., 2026). AE-related costs and disutilities were assumed to occur only once during the first model cycle and were applied as one-time events. To account for the time value of money and quality of life adjustments, costs and utilities were discounted at an annual rate of 4.5% (Wu and Liu, 2026). A comprehensive summary of all model input parameters is provided in Table 2.

**TABLE 2:** Key model inputs Costs, Utility estimates and other parameters.

Parameter	Distribution	Values (range)	References
Treatment costs			
Fuzuloparib (per 50 mg)	Gamma	¥51.6 (38.7–64.5)	Chinese Drug Price of Drug Centralized Bid Procurement. (2026)
Apatinib (per 250 mg)	Gamma	¥93.16 (69.87–116.45)	Chinese Drug Price of Drug Centralized Bid Procurement. (2026)
Capecitabine (per 500 mg)	Gamma	¥1.98 (1.485–2.475)	Chinese Drug Price of Drug Centralized Bid Procurement. (2026)
Vinorelbine (per 10 mg)	Gamma	¥51.12 (38.34–63.9)	Chinese Drug Price of Drug Centralized Bid Procurement. (2026)
Subsequent treatment cost in the FA group (per cycle)	Gamma	¥3,362.3 (2,521.725 to 4,202.875)	Estimated based on the FABULOUS trial and the Yaozh database, and the detailed calculation procedures are presented in Supplementary Table S2
Subsequent treatment cost in the FM group (per cycle)	Gamma	¥4,537.45 (3,403.088 to 5,671.813)	Estimated based on the FABULOUS trial and the Yaozh database, and the detailed calculation procedures are presented in Supplementary Table S2
Subsequent treatment cost in the SC group (per cycle)	Gamma	¥6,736.1 (5,052.075 to 8,420.125)	Estimated based on the FABULOUS trial and the Yaozh database, and the detailed calculation procedures are presented in Supplementary Table S2
Administration (per cycle)	Gamma	¥169.61 (127.21–212.01)	Lang et al. (2024)
Follow-up	Gamma	¥839.04 (629.28–1,048.8)	Lang et al. (2024)
palliative care	Gamma	¥12,757.69 (9,568.27 to 15,947.11)	Lang et al. (2024)
AEs unit costs			
Decreased white blood cells	Gamma	¥3,177.27 (2,382.95 to 3,971.59)	Kang et al. (2026)
Neutrophil count decreased	Gamma	¥2,947.46 (2,210.60 to 3,684.33)	Zhu et al. (2026)
Anemia	Gamma	¥3,634.20 (2,725.65 to 4,542.75)	Zhu et al. (2026)
Platelet count decreased	Gamma	¥24,287.85 (18,215.89 to 30,359.81)	Zhu et al. (2026)
Utility estimates			
PFS state	Beta	0.66 (0.495–0.825)	Schwarz et al. (2022)
PD state	Beta	0.44 (0.33–0.55)	Schwarz et al. (2022)
Disutility estimates			
Decreased white blood cells	Beta	−0.09 (−0.1125 to −0.0675)	Kang et al. (2026)
Neutrophil count decreased	Beta	−0.13 (−0.1625 to −0.0975)	Zhu et al. (2026)
Anemia	Beta	−0.07 (−0.0875 to −0.0525)	Zhu et al. (2026)
Platelet count decreased	Beta	−0.11 (−0.1375 to −0.0825)	Zhu et al. (2026)
Other parameters			
Body surface area	Normal	1.60 m^2^ (1.20–2.00)	Estimated based on the FABULOUS trial and References (National Physical Fitness Monitoring Center, 2026)

In this table, the costs of AEs presented were paid on a per-event basis. Drug prices were obtained from the Yaozh database, with the median bid-winning prices used when multiple manufacturers were available. Healthcare resource costs were inflated to 2025 values using the healthcare-specific Consumer Price Index.

Abbreviations: AEs adverse events, PFS progression-free survival, PD progressed disease.

### Analyses

In the base-case analysis, we conducted an assessment of the incremental cost-utility ratio (ICUR) to determine the additional cost per QALY gained between the three treatment regimens. A treatment strategy is defined as cost-effective if its ICUR was below the established willingness-to-pay (WTP) threshold. In this analysis, the WTP threshold was set at twice the *per capita* gross domestic product (GDP) (Wu and Liu, 2026). For the year 2025, this threshold was estimated to 199,330 Chinese Yuan (CNY), which was applied as the reference threshold for determining cost-utility from the Chinese healthcare system perspective.

To ensure the reliability of our findings, we conducted a series of uncertainty analyses, including one-way deterministic sensitivity analyses (DSA), probabilistic sensitivity analyses (PSA) and Scenario analyses. In the DSA, we examined the impact of individual input uncertainties on the ICUR. The annual discount rate ranged from 0% to 5%, while other model inputs were varied within the reported 95% confidence intervals (CI) or reasonable ranges (±25% of the base-case value). For the PSA, we employed Monte Carlo simulations with 10,000 iterations, simultaneously sampling key parameters based on pre-specified probability distributions. Costs were assigned Gamma distributions, and incidence rates of adverse events (AEs) and utilities were sampled with Beta distributions (Briggs et al., 2012). To provide a comprehensive understanding of the treatment strategy’s cost-utility at different thresholds, we generated cost-effectiveness acceptability curves (CEAC) and scatter plots. These visual representations allowed us to assess the likelihood of the treatment strategy being considered “cost-effective” across a range of thresholds. Scenario analyses and cost-threshold analyses were conducted to further evaluate the robustness of the base-case results under alternative structural. In Scenario 1, the distribution of subsequent treatment categories was standardized across all treatment arms by assuming equal proportions (25% each) of chemotherapy, endocrine therapy, immunotherapy, and targeted therapy among patients receiving subsequent treatment. Accordingly, the expected subsequent treatment cost was recalculated using this equal distribution, resulting in a uniform weighted-average cost of ¥2,505.74 for all treatment arms. The proportions of patients receiving subsequent treatment in each treatment arm remained unchanged. This scenario was designed to evaluate whether differences in the composition of post-progression treatment influenced the results. In Scenario 2, all patients were assumed to receive subsequent treatment after disease progression, while the treatment composition within each arm remained consistent with that reported in the FABULOUS trial. This scenario evaluated the impact of the probability of receiving subsequent treatment on the model outcomes. In Scenario 3, alternative health utility (PFS: 0.87; PD: 0.71) values reported in a published economic evaluation of patients with metastatic HER2-negative breast cancer were adopted to evaluate the influence of utility assumptions on the results (Zhu et al., 2023). In addition, a cost-threshold analysis was performed by applying a common hypothetical subsequent treatment cost across all treatment arms while keeping all other model parameters unchanged. The corresponding ICURs were calculated over a continuous range of subsequent treatment costs, and the detailed results are presented in the Supplementary Figure S2.

All statistical and economic analyses, including PSM and the cost-utility model, were conducted using R 4.4.2 software relying on the heemod (version 1.1.0), survival (version 3.7–0), and flexsurv packages (version 2.3.2) (R Core Team, 2024; Filipović-Pierucci et al., 2017; Therneau, 2020; Jackson, 2016; Luo et al., 2025; Luo et al., 2026).

## Results

### Curve fitting

**FIGURE 2:**
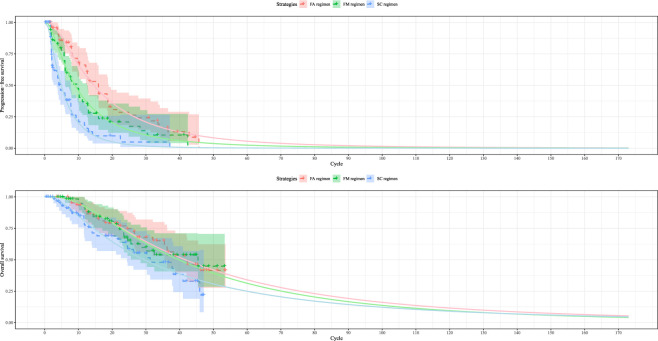
Reconstructed Kaplan-Meier survival curve and the projected OS and PFS curve. Abbreviations: FA Fuzuloparib plus Apatinib, FM Fuzuloparib monotherapy, SC standard chemotherapy. Notes: Each cycle of the x-axis is 3 weeks.

Based on AIC and BIC, the lognormal distribution provided the best fit for both OS and PFS in all treatment groups. Using the selected parametric functions, reconstructed Kaplan–Meier curves were generated, and long-term PFS and OS trajectories were projected to facilitate comparison, as illustrated in Figure 2. The corresponding scale and shape parameters for the fitted survival curves of all treatment strategies are reported in Table 1. Results from alternative parametric distributions, along with their respective fitted curves, are presented in Supplementary Figure S1, and detailed parameter estimates for these models are provided in Supplementary Table S3.

### Base-case analysis

In the base-case analysis, the total costs associated with the three treatment strategies were ¥266,989.27 for the FA group, ¥230,151.42 for the FM group, and ¥188,774.18 for the SC group. Compared with SC, both FA and FM groups incurred higher total costs. When stratified by health state, costs in SC group were primarily incurred during the PD state. In the FA group, the PD state accounted for ¥57,858.46 compared with ¥209,130.81 in the PFS state. In the FM group, costs during the PD state reached ¥107,600.24, whereas costs during the PFS state were ¥122,551.18. For the SC group, the corresponding costs were ¥163,828.68 in the PD state and ¥24,945.50 in the PFS state.

In terms of utility, the FA group generated the highest health benefit with a total of 1.5669 QALYs, followed by the FM group with 1.4128 QALYs and the SC group with 1.1477 QALYs. Specifically, QALYs accrued during the PFS state were 0.7858, 0.5231, and 0.2641 for the FA, FM, and SC groups, respectively, whereas the corresponding QALYs during the PD state were 0.7811, 0.8897, and 0.8836.

Compared with the SC group, the FA group yielded an ICUR of ¥186,581.80 per QALY gained, while the FM group produced an ICUR of ¥156,081.63 per QALY gained. Furthermore, when the FA group was compared directly with the FM group, FA was associated with higher total costs and greater QALYs, yielding an ICUR of ¥239,051.59 per QALY. At a WTP threshold of ¥199,330 per QALY, both FA and FM were below the threshold compared with SC. Nevertheless, compared with the FM group, the FA group was not cost-effective at the WTP threshold of ¥199,330 per QALY. The results of the cost-utility analysis are summarized in Table 3.

**TABLE 3:** Results of the base-case analysis.

Outcomes	FA group	FM group	SC group
Total costs (¥)	266,989.27	230,151.42	188,774.18
PFS state	209,130.81	122,551.18	24,945.50
PD state	57,858.46	107,600.24	163,828.68
Total utilities (QALY)	1.5669	1.4128	1.1477
PFS state	0.7858	0.5231	0.2641
PD state	0.7811	0.8897	0.8836
ICUR (vs. SC)	¥186,581.80/QALY	¥156,081.63/QALY	—
ICUR (vs. FM)	¥239,051.59/QALY	—	—

Abbreviation: QALY quality-adjusted life-year, ICUR incremental cost-utility ratio, FA Fuzuloparib plus Apatinib, FM Fuzuloparib monotherapy, SC standard chemotherapy, PFS progression-free survival, PD progressed disease

### One-way sensitivity analysis

**FIGURE 3:**
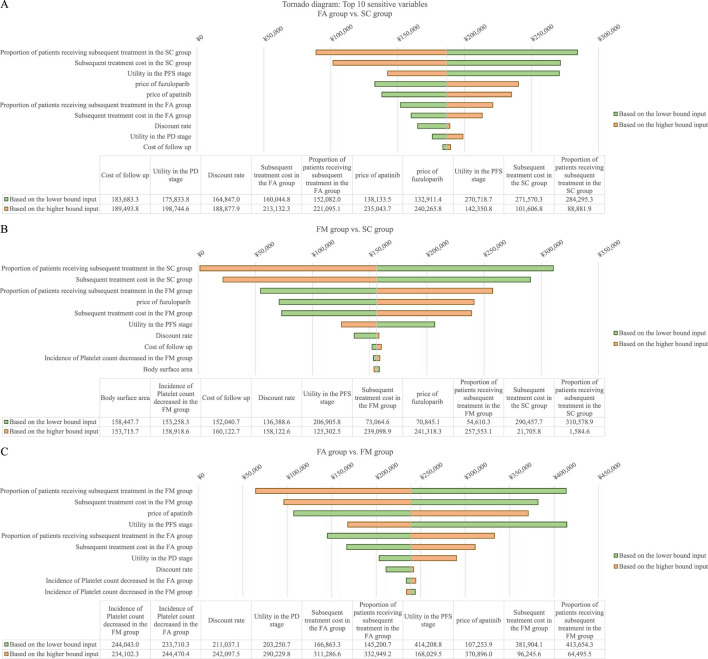
Tornado diagram of one-way sensitivity analysis results. **(A)**. The tornado diagram of the ICUR of FA group versus SC group. **(B)**. The tornado diagram of the ICUR of FM group versus SC group. **(C)**. The tornado diagram of the ICUR of FA group versus FM group. Notes: The x--axis represents the incremental cost -utility ratio. Abbreviations: FA, Fuzuloparib plus Apatinib; FM, Fuzuloparib monotherapy; SC, standard chemotherapy; PFS, progression-free survival; PD, progressed disease.

The results of the one-way sensitivity analyses are presented in Figure 3. Across all pairwise comparisons, parameters related to post-progression treatment were the primary drivers of ICUR uncertainty. In particular, the proportion of patients receiving subsequent treatment and the corresponding subsequent treatment costs exerted the greatest influence on the ICURs. Utility in the PFS state was also among the most influential parameters in all comparisons.

For the comparison between FA and SC, the ICUR was most sensitive to the proportion of patients receiving subsequent treatment in the SC group, followed by the subsequent treatment cost in the SC group, utility in the PFS state, the price of fuzuloparib, and the price of apatinib (Figure 3A). In the comparison between FM and SC, similar findings were observed, with the proportion and cost of subsequent treatment in the SC group remaining the most influential variables, followed by the proportion of patients receiving subsequent treatment in the FM group, the price of fuzuloparib, and the subsequent treatment cost in the FM group (Figure 3B). For the comparison between FA and FM, model outcomes were primarily driven by the proportion of patients receiving subsequent treatment in the FM group, subsequent treatment cost in the FM group, the price of apatinib, utility in the PFS state, and the proportion of patients receiving subsequent treatment in the FA group (Figure 3C).

Notably, variation in several key parameters resulted in ICURs crossing the Chinese WTP threshold of ¥199,330/QALY, particularly parameters related to post-progression treatment, PFS utility and drug price. ICUR results were sensitive to assumptions regarding subsequent treatment patterns, utility, and drug prices.

### Probabilistic sensitivity analysis

**FIGURE 4:**
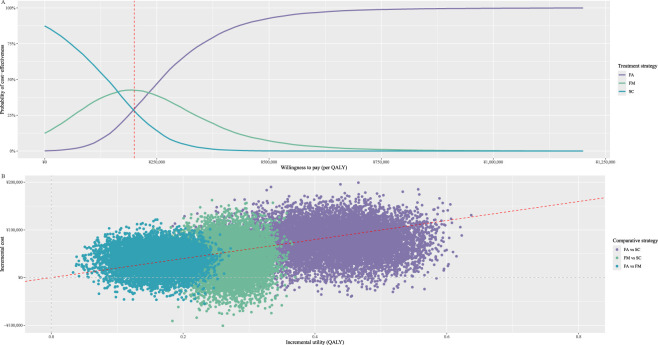
The plot output of probabilistic sensitivity analysis. **(A)**. The cost-utility acceptable curve of FA, FM and SC group. **(B)**. The incremental cost-utility scatter plot for FA vs. SC, FM vs. SC, and FA vs. FM comparisons. In the cost-utility acceptable curve, the y-axis indicates the likelihood that a regimen is cost-effective across the WTP threshold (x-axis). The red dashed line represents the WTP threshold. The monetary unit is Chinese Yuan; In the incremental cost-utility scatter plot, each dot represents one output. The red dashed line represents the predefined WTP threshold. Abbreviations: FA, Fuzuloparib plus Apatinib; FM, Fuzuloparib monotherapy; SC, standard chemotherapy; QALY, quality-adjusted life-year.

The results of the probabilistic sensitivity analysis are presented using CEAC and incremental cost-utility scatter plots (Figure 4). At the Chinese WTP threshold of ¥199,330 per QALY, the probabilities of the FA, FM, and SC regimens being cost-effective were 29.15%, 42.72%, and 28.13%, respectively.

As shown by the CEAC, SC was the preferred strategy at low WTP thresholds. As the WTP threshold increased, the probability of FM being the most cost-effective strategy initially increased and reached its maximum around the Chinese WTP threshold. With further increases in the WTP threshold, the probability of FA being the optimal strategy increased steadily, whereas the probabilities for FM and SC gradually declined.

The incremental cost-utility scatter plots demonstrated that most simulations for both FA versus SC, FM versus SC, and FA versus FM were distributed in the northeast quadrant, indicating that both FA and FM were generally associated with higher costs and greater health benefits than SC, and that FA was likewise associated with higher costs and greater health benefits than FM. Most simulated ICERs were distributed around the WTP threshold, reflecting considerable uncertainty in the economic outcomes under parameter variation.

### Scenario analysis

To further explore the influence of key structural assumptions on the economic outcomes, three scenario analyses were conducted (Table 4). The model structure, time horizon, discount rate, and all other model parameters remained unchanged in all scenario analyses.

**TABLE 4:** Results of scenario analyses.

Analysis	Comparison	Incremental cost (¥)	Incremental QALYs	ICUR (¥/QALY)	Cost-effective at ¥199,330/QALY
Base case	FA vs. SC	78,215.09	0.4192	186,581.80	Yes
	FM vs. SC	41,377.24	0.2651	156,081.63	Yes
	FA vs. FM	36,837.85	0.1541	239,051.59	No
Scenario 1 Standardized subsequent treatment composition	FA vs. SC	156,363.75	0.4192	373,018.47	No
	FM vs. SC	91,446.88	0.2651	344,952.40	No
	FA vs. FM	64,916.87	0.1541	421,305.55	No
Scenario 2 100% subsequent treatment	FA vs. SC	48,709.15	0.4192	116,195.49	Yes
	FM vs. SC	22,189.29	0.2651	83,733.17	Yes
	FA vs. FM	26,519.86	0.1541	172,095.13	Yes
Scenario 3 Alternative utility	FA vs. SC	78,215.09	0.5220	149,823.56	Yes
	FM vs. SC	41,377.24	0.3517	117,649.25	Yes
	FA vs. FM	36,837.85	0.1703	216,311.51	No

In Scenario 1, the distribution of subsequent treatment categories was standardized across all treatment arms by assuming equal proportions of chemotherapy, endocrine therapy, immunotherapy, and targeted therapy among patients receiving subsequent treatment. Under this assumption, the ICURs increased substantially for all pairwise comparisons. Compared with the base-case analysis, the ICURs for FA versus SC, FM versus SC, and FA versus FM increased to ¥373,018.47/QALY, ¥344,952.40/QALY, and ¥421,305.55/QALY, respectively, and none of the three strategies remained cost-effective at the current Chinese WTP threshold.

In Scenario 2, all patients were assumed to receive subsequent treatment after disease progression while maintaining the treatment composition reported in the FABULOUS trial. Under this assumption, the ICURs decreased to ¥116,195.49/QALY for FA versus SC, ¥83,733.17/QALY for FM versus SC, and ¥172,095.13/QALY for FA versus FM. Consequently, all three comparisons were considered cost-effective at the current Chinese WTP threshold.

In Scenario 3, alternative published utility values were applied. Although the incremental QALYs increased across all comparisons, the resulting ICURs changed only modestly compared with the base-case analysis. Both FA and FM remained cost-effective compared with SC, whereas FA was still not cost-effective compared with FM.

## Discussion

Breast cancer remains the most frequently diagnosed malignancy worldwide and a leading cause of cancer-related mortality, with a particularly substantial disease burden in China and other low- and middle-income countries (Fan et al., 2014; Zhou et al., 2024). Patients with BRCA-mutated breast cancer often experience poorer long-term outcomes (Kuchenbaecker et al., 2017). These patients are associated with substantial clinical and economic burden due to prolonged treatment and multiple lines of therapy, and increased healthcare utilization (Sun et al., 2018). The introduction of PARP inhibitors has significantly reshaped the therapeutic landscape for this molecularly defined subgroup by exploiting defects in homologous recombination repair through synthetic lethality. However, these clinical benefits are inevitably accompanied by increased treatment costs, making economic evaluation essential for supporting reimbursement decisions and efficient healthcare resource allocation. Based on clinical evidence from the phase III FABULOUS trial, this study evaluated the long-term cost-utility of fuzuloparib plus apatinib, fuzuloparib monotherapy, and standard chemotherapy in patients with HER2-negative metastatic breast cancer harboring germline BRCA1/2 mutations from the perspective of the Chinese healthcare system.

Three principal findings emerged from this study. First, both FA and FM were cost-effective compared with SC at the current Chinese WTP threshold. Second, although FA generated the greatest health benefit among the three treatment strategies, it was not cost-effective compared with FM because the additional health benefits were achieved at an incremental cost exceeding the WTP threshold. Third, subsequent treatment patterns after disease progression were the primary contributors to decision uncertainty, emphasizing the importance of accurately characterizing post-progression management in economic evaluations.

The base-case results demonstrated that both the FA and FM regimens significantly improved health outcomes compared with standard chemotherapy, primarily through prolonged progression-free survival and increased QALY accumulation. Among the three treatment strategies, FA achieved the highest QALYs, followed by FM and SC. Compared with SC, both FA and FM produced ICURs below the Chinese WTP threshold, indicating cost-effective treatment options under current WTP threshold. However, the comparison between FA and FM yielded an ICUR above the WTP threshold, suggesting that although the addition of apatinib further improved clinical outcomes, the magnitude of the incremental benefit was insufficient to justify the additional cost under current WTP threshold from the perspective of the Chinese healthcare system.

An important finding of this study is that the distribution of healthcare expenditures differed substantially across treatment strategies. Delayed disease progression shifted a larger proportion of total costs toward the progression-free state because patients remained on maintenance therapy for longer durations. Although this delay reduced downstream expenditures associated with disease progression and subsequent treatment, these savings only partially compensated for the additional acquisition costs of combination therapy. Consequently, under current WTP, the incremental health benefits associated with FA were insufficient to offset its higher costs relative to FM.

Because treatment patterns after disease progression vary considerably in clinical practice, subsequent treatment was modeled according to the treatment distribution reported in the FABULOUS trial. Both the proportion of patients receiving subsequent therapy and the relative distribution of chemotherapy, endocrine therapy, immunotherapy, and targeted therapy were incorporated into the model. Representative regimens recommended by the CSCO guidelines were selected for each treatment category, and expected subsequent treatment costs were estimated using a weighted approach. This strategy allows the model to better reflect routine clinical practice while maintaining transparency regarding model assumptions.

The sensitivity analyses further demonstrated that assumptions regarding subsequent treatment influenced the economic outcomes. Across all treatment comparisons, the proportion of patients receiving subsequent therapy and the associated subsequent treatment costs consistently ranked among the most influential parameters. Utility associated with the progression-free state also exerted a considerable impact on the ICUR estimates. These findings indicate that both treatment sequencing after disease progression and health-related quality of life are key determinants of the long-term economic value of maintenance therapy. Probabilistic sensitivity analysis also demonstrated considerable decision uncertainty. At the Chinese WTP threshold of ¥199,330 per QALY, FM had the highest probability of being the most cost-effective strategy, whereas FA and SC showed comparable probabilities. As the WTP threshold increased, however, the probability of FA being the preferred strategy increased progressively and ultimately approached 100%. These findings suggest that the preferred strategy is sensitive to parameter uncertainty and healthcare resource availability.

The scenario analyses further reinforced our principal finding that assumptions regarding post-progression treatment represent a major source of structural uncertainty in the present model. Together with the deterministic sensitivity analysis, these results demonstrate that both the composition of subsequent treatment and the proportion of patients receiving subsequent therapy can substantially influence the estimated economic outcomes. The additional cost-threshold analysis further complements the scenario analyses by quantifying the influence of subsequent treatment costs on the cost-utility results. Rather than relying on discrete scenario assumptions, this analysis identified the threshold subsequent treatment cost at which the conclusions changed. Future studies should prioritize the collection of more detailed real-world evidence on subsequent treatment patterns to further reduce uncertainty.

Recently, Fu et al. published a related cost-effectiveness analysis of fuzuloparib, with or without apatinib, based on the same FABULOUS trial, but using a 20-year time horizon rather than the 10-year horizon adopted in our model (Fu et al., 2026). Their analysis concluded that neither fuzuloparib monotherapy nor fuzuloparib plus apatinib was cost-effective relative to standard chemotherapy, and that fuzuloparib plus apatinib is more cost-effective than fuzuloparib alone. This conclusion contrasts with the findings of our study. This discrepancy may partly arise from substantial differences between the two models in estimated subsequent-treatment costs, which are likely related both to differing assumptions regarding post-progression treatment patterns and to the different time horizons adopted, since a longer horizon changes the duration and composition of subsequent therapy that patients are assumed to receive. The two studies also used different sources for health utility values. Notably, our one-way sensitivity analysis identified subsequent-treatment cost and utility inputs among the most influential parameters on the ICUR, indicating that comparatively modest differences in these inputs could be sufficient to alter the conclusions. The two studies are therefore best regarded as complementary rather than conflicting, reflecting different modeling assumptions, and warranting further validation with real-world evidence.

This study provides several important policy implications. First, although both fuzuloparib and apatinib are listed in the National Reimbursement Drug List (NRDL), neither is currently reimbursed for breast cancer indications. The present findings suggest that PARP inhibitor-based maintenance therapy represents a cost-effective alternative to standard chemotherapy under current pricing conditions. However, from the perspective of selecting between the two PARP inhibitor-based strategies, the additional cost associated with adding apatinib is not currently justified by the incremental health benefit. Future reductions in the price of apatinib or optimized treatment strategies may further improve the economic attractiveness of FA. Second, our findings highlight the importance of standardizing post-progression treatment pathways in clinical practice, as assumptions regarding subsequent treatment substantially influenced the economic outcomes. Finally, value-based patient selection strategies, such as those guided by biomarkers or clinical characteristics, may enhance the efficiency of resource allocation by identifying patients most likely to benefit from combination maintenance therapy.

However, several limitations should be acknowledged. First, the use of reconstructed individual patient data introduces potential uncertainty. To address this issue, we applied Guyot’s algorithm, which is widely used in health economic evaluations and has been shown to provide reliable reconstruction of survival data (Saluja et al., 2019). Second, utility values were obtained from published literature rather than directly measured in the FABULOUS trial, which may limit their applicability to the study population. Third, although subsequent treatment patterns were modeled using the published FABULOUS trial data together with guideline-recommended representative regimens, patient-level information regarding the exact sequence and duration of multiple subsequent treatment lines was unavailable. Consequently, weighted average costs were used to approximate subsequent treatment expenditures, which may still introduce uncertainty into the model. Finally, subgroup analyses were not performed in this study. Although the FABULOUS trial reported HRs for PFS across different subgroups, corresponding HRs for OS were not fully available. Moreover, survival modeling based on curve extrapolation requires subgroup-specific survival curves or individual patient data, which were not accessible. The absence of such data limited our ability to conduct robust subgroup analyses. While some studies have attempted to approximate subgroup outcomes by adjusting HRs, this approach relies on applying subgroup HRs to the overall population survival curves rather than true subgroup-specific data. Therefore, we considered this assumption insufficiently robust and did not adopt this approach in the present analysis.

## Conclusion

Both FA and FM were cost-effective compared with standard chemotherapy from the perspective of the Chinese healthcare system. However, FA was not cost-effective relative to FM at the current Chinese WTP threshold, indicating that the additional clinical benefit associated with apatinib does not fully justify its incremental cost under current pricing conditions. Future studies incorporating mature survival data, real-world treatment patterns, and updated drug prices will further clarify the long-term economic value of PARP inhibitor-based maintenance therapy.

## Data Availability

The original contributions presented in the study are included in the article/Supplementary Material, further inquiries can be directed to the corresponding author.
