# Supplementary material for: Fuzuloparib with or without apatinib in patients with HER2-negative metastatic breast cancer with germline BRCA1/2 mutations: a cost-utility analysis
Source: Front Pharmacol. 2026 Jul 29;17:1897783. doi: 10.3389/fphar.2026.1897783 (PMC13461066; doi:10.3389/fphar.2026.1897783)
Supplement: Supplementary file 1 [file DataSheet1.docx]

| Supplementary Table 1 The CHEERS 2022 checklist | | | |
| --- | --- | --- | --- |
| Topic | **Item No** | **Guidance for reporting** | **Article section** |
| Title | | | |
| Title | 1 | Identify the study as an economic evaluation and specify the interventions being compared. | Title |
| Abstract | | | |
| Abstract | 2 | Provide a structured summary that highlights context, key methods, results, and alternative analyses. | Abstract |
| Introduction | | | |
| Background and objectives | 3 | Give the context for the study, the study question, and its practical relevance for decision making in policy or practice. | Introduction |
| Methods | | | |
| Health economic analysis plan | 4 | Indicate whether a health economic analysis plan was developed and where available. | Materials and Methods |
| Study population | 5 | Describe characteristics of the study population (such as age range, demographics, socioeconomic, or clinical characteristics). | Materials and Methods- Clinical data |
| Setting and location | 6 | Provide relevant contextual information that may influence findings. | Materials and Methods |
| Comparators | 7 | Describe the interventions or strategies being compared and why chosen. | Materials and Methods- Treatment regimens and Resource use |
| Perspective | 8 | State the perspective(s) adopted by the study and why chosen. | Materials and Methods- Model structure |
| Time horizon | 9 | State the time horizon for the study and why appropriate. | Materials and Methods- Model structure |
| Discount rate | 10 | Report the discount rate(s) and reason chosen. | Materials and Methods-Analyses |
| Selection of outcomes | 11 | Describe what outcomes were used as the measure(s) of benefit(s) and harm(s). | Materials and Methods-Analyses |
| Measurement of outcomes | 12 | Describe how outcomes used to capture benefit(s) and harm(s) were measured. | Materials and Methods- Model structure, Analyses |
| Valuation of outcomes | 13 | Describe the population and methods used to measure and value outcomes. | Materials and Methods- Model structure |
| Measurement and valuation of resources and costs | 14 | Describe how costs were valued. | Materials and Methods-Costs and utilities |
| Currency, price date, and conversion | 15 | Report the dates of the estimated resource quantities and unit costs, plus the currency and year of conversion. | Materials and Methods-Costs and utilities |
| Rationale and description of model | 16 | If modelling is used, describe in detail and why used. Report if the model is publicly available and where it can be accessed. | Materials and Methods- Model structure |
| Analytics and assumptions | 17 | Describe any methods for analysing or statistically transforming data, any extrapolation methods, and approaches for validating any model used. | Materials and Methods-Clinical data |
| Characterizing heterogeneity | 18 | Describe any methods used for estimating how the results of the study vary for subgroups. | Not applicable |
| Characterizing distributional effects | 19 | Describe how impacts are distributed across different individuals or adjustments made to reflect priority populations. | Not applicable |
| Characterizing uncertainty | 20 | Describe methods to characterise any sources of uncertainty in the analysis. | Materials and Methods-Analyses |
| Approach to engagement with patients and others affected by the study | 21 | Describe any approaches to engage patients or service recipients, the general public, communities, or stakeholders (such as clinicians or payers) in the design of the study. | Not applicable |
| Results | | | |
| Study parameters | 22 | Report all analytic inputs (such as values, ranges, references) including uncertainty or distributional assumptions. | Table 1 and Table 2 |
| Summary of main results | 23 | Report the mean values for the main categories of costs and outcomes of interest and summarise them in the most appropriate overall measure. | Results-Cost-effectiveness analysis; Table 3 |
| Effect of uncertainty | 24 | Describe how uncertainty about analytic judgments, inputs, or projections affect findings. Report the effect of choice of discount rate and time horizon, if applicable. | Results-Sensitivity analysis and additional analysis;  Figure 3, Table 4 |
| Effect of engagement with patients and others affected by the study | 25 | Report on any difference patient/service recipient, general public, community, or stakeholder involvement made to the approach or findings of the study | Not applicable |
| Discussion | | | |
| Study findings, limitations, generalizability, and current knowledge | 26 | Report key findings, limitations, ethical or equity considerations not captured, and how these could affect patients, policy, or practice. | Discussion |
| Other relevant information | | | |
| Source of funding | 27 | Describe how the study was funded and any role of the funder in the identification, design, conduct, and reporting of the analysis | Declarations- Funding |
| Conflicts of interest | 28 | Report authors conflicts of interest according to journal or International Committee of Medical Journal Editors requirements. | Declarations- Conflict of interest statement: |
| For consistency, the CHEERS Statement checklist format is based on the format of the CONSORT 2 statement checklist^1^ | | | |

| **Supplementary Table 2**. Assumptions for Subsequent Treatment Costs After Disease Progression | | | | | |
| --- | --- | --- | --- | --- | --- |
| Treatment category | Representative regimen and dosing strategy | Cycle cost | FA | FM | SC |
| Chemotherapy | Capecitabine or Vinorelbine (capecitabine: 1000–1250 mg/m² administered orally twice daily on days 1–14 of each 21-day cycle; vinorelbine: 25–30 mg/m² intravenously on days 1 and 8 of each 21-day cycle.) | ¥356.26 | 83.33% | 83.78% | 40% |
| Endocrine therapy | Palbociclib plus Anastrozole (palbociclib: 125 mg orally once daily for 21 days, followed by 7 days off treatment; anastrozole: 1 mg orally once daily) | ¥214.43 | 33.33% | 40.54% | 22.5% |
| Immunotherapy | Toripalimab plus Nab-paclitaxel (toripalimab: 240 mg  intravenously on day 1 of each 21-day cycle; nab-paclitaxel: 260 mg/m² intravenously on days 1 of each 21-day cycle) | ¥2,950.65 | 13.33% | 21.62% | 12.5% |
| Targeted therapy | Fuzuloparib monotherapy (fuzuloparib: 150 mg orally twice daily) | ¥6,501.6 | 40% | 54.05% | 95% |
| Weighted subsequent treatment cycle cost | — | — | ¥3,362.3 | ¥4,537.45 | ¥6,736.1 |
| Patients receiving subsequent treatment | — | — | 42.9% | 55.2% | 60.6% |
| **Notes:** Subsequent treatment categories and treatment proportions were obtained from the FABULOUS trial. The proportions shown for each treatment category were calculated among patients who received subsequent therapy. Representative regimens for each treatment category were selected according to the CSCO guidelines. The expected subsequent treatment cost for each treatment arm was estimated using a two-stage weighted approach by combining (1) the probability of receiving subsequent treatment and (2) the weighted average cost of different treatment categories.  **Drug price:** Fuzuloparib (¥51.6 per 50 mg), Capecitabine (¥1.98 per 500 mg), Vinorelbine (¥51.12 per 10 mg), Toripalimab (¥1884.86 per 240 mg), Nab-paclitaxel (¥256.2 per 100mg), Anastrozole (¥1.104 per 1 mg), Palbociclib (¥12.143 per 125 mg).  **Abbreviations: *FA*** Fuzuloparib plus Apatinib, ***FM*** Fuzuloparib monotherapy, ***SC*** standard chemotherapy, ***CSCO*** Chinese Society of Clinical Oncology | | | | | |

| Treatment arm | Distribution | Shape parameters | Value | L95% | U95% | SE | AIC | BIC |
| --- | --- | --- | --- | --- | --- | --- | --- | --- |
| PFS: FA regimen | Log-logistic | shape | 1.956 | 1.551 | 2.467 | 0.232 | 376.8006 | 381.2976 |
|  |  | scale | 14.872 | 11.801 | 18.742 | 1.755 |  |  |
|  | Weibull | shape | 1.39 | 1.117 | 1.729 | 0.155 | 378.1065 | 382.6035 |
|  |  | scale | 21.371 | 17.383 | 26.273 | 2.252 |  |  |
|  | Exponential | rate | 0.04691 | 0.03525 | 0.06244 | 0.00684 | 383.5882 | 385.8367 |
|  | Lognormal | meanlog | 2.6817 | 2.4473 | 2.9162 | 0.1196 | 376.0413 | 380.5383 |
|  |  | sdlog | 0.8871 | 0.7244 | 1.0862 | 0.0917 |  |  |
|  | Gompertz | shape | 0.0249 | -0.0000804 | 0.05 | 0.0128 | 382.0327 | 386.5297 |
|  |  | rate | 0.0336 | 0.021 | 0.0537 | 0.00806 |  |  |
|  | Gamma | shape | 1.7598 | 1.2401 | 2.4973 | 0.3143 | 376.8001 | 381.2971 |
|  |  | rate | 0.0901 | 0.0578 | 0.1404 | 0.0204 |  |  |
| PFS: FM regimen | Log-logistic | shape | 1.785 | 1.421 | 2.243 | 0.208 | 357.2689 | 361.6783 |
|  |  | scale | 8.953 | 7.005 | 11.444 | 1.121 |  |  |
|  | Weibull | shape | 1.181 | 0.957 | 1.457 | 0.127 | 363.5265 | 367.9358 |
|  |  | scale | 13.889 | 10.967 | 17.59 | 1.674 |  |  |
|  | Exponential | rate | 0.073 | 0.0553 | 0.0963 | 0.0103 | 363.7265 | 365.9312 |
|  | Lognormal | meanlog | 2.198 | 1.951 | 2.445 | 0.126 | 356.0013 | 360.4107 |
|  |  | sdlog | 0.962 | 0.788 | 1.175 | 0.098 |  |  |
|  | Gompertz | shape | 0.0015 | -0.03 | 0.033 | 0.0161 | 365.7179 | 370.1273 |
|  |  | rate | 0.072 | 0.0482 | 0.1077 | 0.0148 |  |  |
|  | Gamma | shape | 1.4321 | 1.0176 | 2.0154 | 0.2497 | 361.8347 | 366.244 |
|  |  | rate | 0.1102 | 0.0705 | 0.1724 | 0.0252 |  |  |
| PFS: SC regimen | Log-logistic | shape | 1.927 | 1.537 | 2.415 | 0.222 | 274.9326 | 279.3119 |
|  |  | scale | 4.644 | 3.635 | 5.933 | 0.58 |  |  |
|  | Weibull | shape | 1.166 | 0.956 | 1.422 | 0.118 | 285.5701 | 289.9494 |
|  |  | scale | 7.69 | 5.998 | 9.86 | 0.975 |  |  |
|  | Exponential | rate | 0.133 | 0.0999 | 0.177 | 0.0194 | 285.6535 | 287.8431 |
|  | Lognormal | meanlog | 1.5854 | 1.355 | 1.8157 | 0.1175 | 271.492 | 275.8713 |
|  |  | sdlog | 0.8627 | 0.7056 | 1.0548 | 0.0885 |  |  |
|  | Gompertz | shape | -0.00502 | -0.04806 | 0.03802 | 0.02196 | 287.6 | 291.9793 |
|  |  | rate | 0.13695 | 0.09384 | 0.19985 | 0.02641 |  |  |
|  | Gamma | shape | 1.4937 | 1.0596 | 2.1056 | 0.2617 | 282.8955 | 287.2748 |
|  |  | rate | 0.2098 | 0.1343 | 0.3278 | 0.0478 |  |  |
| OS: FA regimen | Log-logistic | shape | 1.963 | 1.428 | 2.699 | 0.319 | 257.9381 | 262.4351 |
|  |  | scale | 41.475 | 31.274 | 55.002 | 5.974 |  |  |
|  | Weibull | shape | 1.677 | 1.214 | 2.316 | 0.276 | 258.5119 | 263.0089 |
|  |  | scale | 51.985 | 39.964 | 67.621 | 6.975 |  |  |
|  | Exponential | rate | 0.01426 | 0.00964 | 0.02111 | 0.00285 | 264.5053 | 266.7538 |
|  | Lognormal | meanlog | 3.73 | 3.424 | 4.036 | 0.156 | 256.5488 | 261.0457 |
|  |  | sdlog | 0.885 | 0.661 | 1.184 | 0.131 |  |  |
|  | Gompertz | shape | 0.03352 | 0.0049 | 0.06214 | 0.0146 | 261.436 | 265.933 |
|  |  | rate | 0.00728 | 0.00337 | 0.01569 | 0.00285 |  |  |
|  | Gamma | shape | 2.13 | 1.3359 | 3.3959 | 0.5069 | 257.787 | 262.284 |
|  |  | rate | 0.0436 | 0.0223 | 0.0853 | 0.0149 |  |  |
| OS: FM regimen | Log-logistic | shape | 2.029 | 1.45 | 2.839 | 0.348 | 235.7274 | 240.1368 |
|  |  | scale | 38.922 | 29.177 | 51.923 | 5.723 |  |  |
|  | Weibull | shape | 1.667 | 1.191 | 2.335 | 0.286 | 238.0354 | 242.4448 |
|  |  | scale | 50.226 | 37.831 | 66.683 | 7.263 |  |  |
|  | Exponential | rate | 0.01437 | 0.00955 | 0.02163 | 0.003 | 243.1509 | 245.3556 |
|  | Lognormal | meanlog | 3.683 | 3.377 | 3.989 | 0.156 | 233.853 | 238.2624 |
|  |  | sdlog | 0.844 | 0.618 | 1.153 | 0.134 |  |  |
|  | Gompertz | shape | 0.02672 | -0.00493 | 0.05836 | 0.01615 | 242.5587 | 246.9681 |
|  |  | rate | 0.00892 | 0.0042 | 0.01893 | 0.00342 |  |  |
|  | Gamma | shape | 2.2417 | 1.361 | 3.6923 | 0.5707 | 236.4981 | 240.9075 |
|  |  | rate | 0.0485 | 0.0237 | 0.0994 | 0.0178 |  |  |
| OS: SC regimen | Log-logistic | shape | 1.666 | 1.226 | 2.263 | 0.261 | 251.4375 | 255.8168 |
|  |  | scale | 29.736 | 21.384 | 41.352 | 5.003 |  |  |
|  | Weibull | shape | 1.4 | 1.03 | 1.91 | 0.22 | 251.2262 | 255.6055 |
|  |  | scale | 39.61 | 29.61 | 53 | 5.89 |  |  |
|  | Exponential | rate | 0.02168 | 0.01476 | 0.03185 | 0.00425 | 253.2204 | 255.4101 |
|  | Lognormal | meanlog | 3.385 | 3.031 | 3.739 | 0.181 | 250.6342 | 255.0135 |
|  |  | sdlog | 1.049 | 0.794 | 1.385 | 0.149 |  |  |
|  | Gompertz | shape | 0.02706 | -0.00411 | 0.05822 | 0.0159 | 252.4613 | 256.8406 |
|  |  | rate | 0.01386 | 0.00693 | 0.02772 | 0.0049 |  |  |
|  | Gamma | shape | 1.6225 | 1.0473 | 2.5137 | 0.3624 | 250.9522 | 255.3315 |
|  |  | rate | 0.0433 | 0.0226 | 0.0831 | 0.0144 |  |  |
| **Abbreviations: *FA*** Fuzuloparib plus Apatinib, ***FM*** Fuzuloparib monotherapy, ***SC*** standard chemotherapy, ***PFS*** progression-free survival, ***OS*** overall survival. | | | | | | | | |

**Supplementary Figure 1.** The fit figures based on other alternative distributions.

***Abbreviations:*** ***FA*** Fuzuloparib plus Apatinib, ***FM*** Fuzuloparib monotherapy, ***SC*** standard chemotherapy.

**Notes:** Each cycle of the x-axis is three weeks.

**Supplementary Figure 2**. Cost-threshold analysis of common subsequent treatment costs.

***Abbreviations:*** ***FA*** Fuzuloparib plus Apatinib, ***FM*** Fuzuloparib monotherapy, ***SC*** standard chemotherapy ***QALY*** quality-adjusted life-year, ***ICUR*** incremental cost-utility ratio, ***WTP*** willingness-to-pay.

**Notes:** This figure illustrates the relationship between the common subsequent treatment cost and the corresponding ICURs for each pairwise treatment comparison. The analysis was conducted by applying a common hypothetical subsequent treatment cost equally across all treatment arms while keeping all other model parameters unchanged. The horizontal dashed line represents the Chinese WTP threshold (¥199,330/QALY). The intersections between each ICUR curve and the WTP threshold indicate the threshold subsequent treatment cost at which the corresponding comparison becomes cost-effective.
